# The Effect of Non-Circadian Photoperiod on Growth and Puberty Onset of Brook Trout *Salvelinus fontinalis* Mitchill

**DOI:** 10.3390/ani11030692

**Published:** 2021-03-05

**Authors:** Katsiaryna Lundova, Jan Matousek, Vlastimil Stejskal

**Affiliations:** Institute of Aquaculture and Protection of Waters, South Bohemian Research Center of Aquaculture and Biodiversity of Hydrocenoses, Faculty of Fisheries and Protection of Waters, University of South Bohemia in České Budějovice, Na Sádkách 1780, 370 05 České Budějovice, Czech Republic; matouj03@frov.jcu.cz (J.M.); stejskal@frov.jcu.cz (V.S.)

**Keywords:** *Salvelinus fontinalis*, photoperiod, growth, sexual maturation, puberty

## Abstract

**Simple Summary:**

There has been intensive research addressing the positive effects of different prolonged photoperiods on wide spectrum of aspects of salmonids aquaculture. The present study was an attempt to assess non-circadian photoperiod regimens on growth and puberty onset of brook trout. We found regimen under which fish was exposed to 48 h of natural ambient photoperiod alternating with 24 h of constant light to be remarkably effective on the delay of gonad development and onset of puberty, enabling fish farmers to fight with negative aspects related to brook trout puberty.

**Abstract:**

The aim of the present study was to assess the effects of a prolonged photoperiod on growth rate and sexual maturation in brook trout *Salvelinus fontinalis.* The task of the experiment was to determine the most effective light regimen capable to minimizing the effects of puberty, including impairment of somatic growth and further general characteristics. In this regard, the studied fish were reared under three photoperiod regimens in which fish were exposed to 24 h continuous light alternating with 24 or 48 h under the ambient photoperiod or 48 h continuous light alternating with a 24 h ambient photoperiod. A control group was reared under the natural ambient photoperiod. Four-hundred and fifty fish with an average initial body weight of 101.3 ± 1.2 g were used for each experimental group (three replicates of each treatment plus control). A statistically lower growth rate showed control groups in both sexes. At the end of the study, control males had an average body weight of 226.6 ± 39.8 g and control females a body weight of 199.8 ± 12.2 g. At the same period, a significantly higher average body weight was found in groups reared 24 h under ambient photoperiod alternating with a 48 h continuous light regime (2CP:1AP) in both sexes (296.56 ± 62.5 g—males, and 239.9 ± 19.2 g—females, respectively). A significantly higher percentage of sexually mature fish was observed in the control group (80% of males and 29% of females, respectively). We found significantly fewer sexually mature females compared to males. The lowest survival was observed in group 2CP:1AP at 92%. It was concluded that regimen under which fish was exposed to 48 h of natural ambient photoperiod alternating with 24 h of constant light (1CP:2AP) lead to the successful delay of gonad development and onset of puberty and increased somatic growth in both sexes.

## 1. Introduction

Brook trout *Salvelinus fontinalis* Mitchill is a North American salmonid that has become popular in Central and Northern European aquaculture in recent decades [1]. It is highly adaptable to a range of aquaculture systems, tolerant to low pH and a wide range of temperatures and produces highly palatable meat [2,3].

Puberty is a physiological process that starts after sex differentiation and determined by the onset of germ cell maturation and full functional differentiation of the germ cell-supporting somatic cells of the gonads. It culminates in the first spermiation and sperm hydration or ovulation, depending on the sex of the individual [4]. Based on the available knowledge about salmonids, it can be assumed that maturation in brook trout may initiate after the summer solstice [5]. The typical spawning period is autumn [6], spawning usually takes place between August and December when water temperatures begin dropping after summer [7]. Based on previous studies, it can be assumed that spawning and optimal growth of brook trout occurred in temperatures as high as 16 °C [8,9].

Despite the success in developing rearing technologies and the growing market interest, early sexual maturation is a major problem in the on-growing industry. Precocious maturation has a negative impact on the growth rate—mature individuals invest heavily in the development of gonads and show negative growth performance [10,11], the immune system—by inability of producing isohemagglutinins, which can leads to the reduction of bactericidal activity [12], and product quality—softer fillets with reduced fat and low slaughter yield [13]. In early puberty, fish show a high growth potential, a factor increasing profitability in the farming industry [14], but somatic weight subsequently stabilizes or decreases, as resources are diverted to gonad formation and gamete production [4].

Sexual maturation of salmonids is controlled by factors including body size, growth rate, and fat deposition [15], and abiotic influences such as temperature, photoperiod, diet, stress, and behavior [16,17]. In vertebrates, the photoperiod has a significant effect on the activity of the brain pituitary–adrenal axis and, as a result, on gonad maturation [18]. Photic signals are transmitted by pineal complex and/or lateral eyes to neuroendocrine neurons, which is subsequently accompanied by the production of gonadotropin-releasing hormone (GnRH) [19]. GnRH, in turn, stimulates the pituitary gland to release two types of gonadotropins—follicle-stimulating hormone (FSH) and luteinizing hormone (LH) [20]. FSH stimulates ovarian follicles to secrete estradiol-17β, which is responsible for stimulating the liver to produce vitellogenin for the initiation of sexual maturation. LH is responsible for the final of maturation [21]. Sexual maturation presents a number of problems that threaten the stability of aquaculture production [19]. Those issues including increased aggression of males [11], reduced fillet yield of males and females [22], decreasing of flesh quality [13], and vulnerability to secondary diseases [3].

Photoperiod manipulation can affect reproduction, growth rate, and feeding of salmonids without a negative outcome [23]. Thus, photoperiod manipulation is used in fish farming to increase efficiency and enable year-round reproduction [24,25]. Currently, there are a number of research papers presenting results that support this assumption. For example, Liu and Duston [26] presented that continuous 24 h light regimen during the fall–winter period can be effective in delaying puberty in Arctic charr (*Salvelinus alpinus*). A similar result was obtained for Atlantic salmon (*Salmo salar*). In that study, the inhibitory effect of continuous 24 h light photoperiod of gonadal development was noted one month after the onset of this light regimen based on histological and hormone analysis [27].

The present study was set up to investigate the effect of non-circadian light regimes on growth and sexual maturation in brook trout. Fish were reared under three different non-circadian photoperiods—24 h of ambient photoperiod alternating with a 24 h of continuous light (1CP:1AP), 24 h of ambient photoperiod alternating with 48 h continuous light (2CP:1AP), and 48 h of natural ambient photoperiod alternating with 24 h of continuous photoperiod (1CP:2AP). The rationale for choosing these regimes was based on the aim to minimize the number of days when fish are exposed to an extended photoperiod and thus contribute to reducing anticipated stress conditions for fish, reducing electricity consumption, and developing a more economically and less labor-intensive strategy for intensive aquaculture. We hypothesize that application of a non-circadian photoperiod will influence the onset of puberty and result in reallocation of energy before and during the spawning period.

## 2. Materials and Methods

### 2.1. Experimental Protocol

The study was carried out from 21 June to 7 November (140 days) at Rybářství Litomyšl Ltd., Litomyšl, Czech Republic.

Randomly collected *S. fontinalis* (*n* = 1800) mean weight 101.3 ± 1.2 g were distributed among twelve outdoor aerated flow-through tanks (water depth 0.85 m, volume 2.5 m^3^) with a flow rate of 3.0–3.5 m^3^ h^−1^. Fish were separated into four groups consisting of 150 specimens in each of three tanks (450 fish per group, three replicates of each treatment). Three treatment tanks were equipped with a system of light-emitting diodes (LED). Two lights, each fitted with an Epistar 50 W LED CO8 (50 W, 4000 Lm, 6000 K), were placed 50 cm above the water level in each tank, producing light intensity of 250–1000 lux as measured with a Hydrolux underwater illumination meter (luxmeter) (BGB Innovation, Grantham, UK) from c. 0.4 m below the water surface.

**Figure 1:**
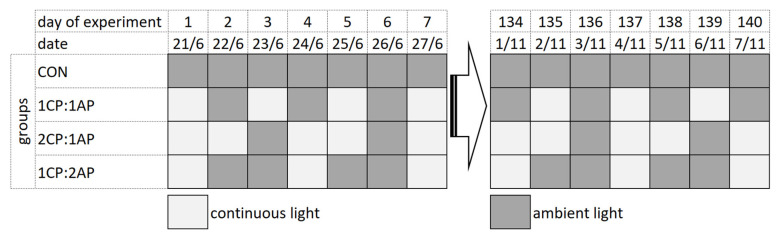
Experimental design for testing non-circadian photoperiod on growth and puberty onset of brook trout *Salvelinus fontinalis*. 2CP:1AP—group exposed to 24 h of ambient photoperiod alternating with a 48 h of continuous light, 1CP:2AP—group exposed to 48 h of ambient photoperiod alternating with a 24 h of continuous light, 1CP:1AP—group exposed to 24 h of ambient photoperiod alternating with 24 h of continuous light, and CON—group exposed to natural ambient photoperiod as a control.

Three groups of fish were exposed to non-circadian photoperiod regimes. Group 1CP:1AP was exposed to 24 h of ambient photoperiod alternating with a 24 h of continuous light for 140 days (70 days of continuous light in total). Group 2CP:1AP was exposed to 24 h of ambient photoperiod alternating with 48 h continuous light (94 days of continuous light in total). Group 1CP:2AP were exposed to 48 h of natural ambient photoperiod alternating with 24 h of a continuous photoperiod (47 days of continuous light in total). A fourth group (CON) was reared throughout 140 days under the natural ambient photoperiod as a control. A schematic representation of the used photoperiod regimes for all experimental groups is presented in Figure 1, which shows the alteration of natural and artificially extended daylight hours throughout the experiment.

**Figure 2:**
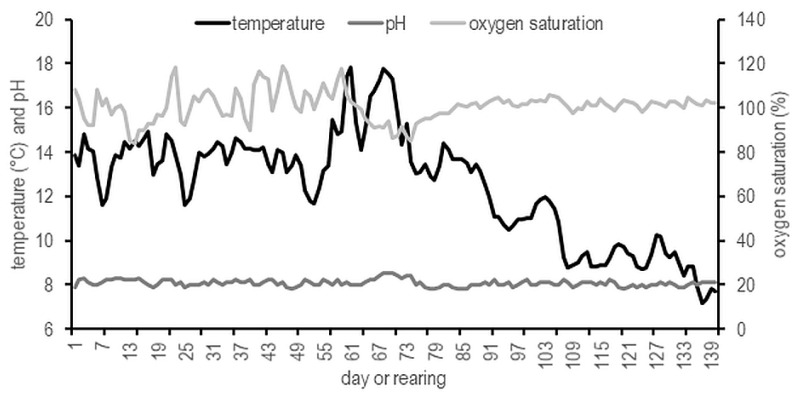
Temperature, pH, and oxygen saturation during testing the non-circadian photoperiod on growth and puberty onset of brook trout *Salvelinus fontinalis*.

Fish were manually fed twice (08:00 and 15:00) a day with fed Biomar EFICO Alpha 756 (protein 39–42%, fat 21–24%, and carbohydrate 21%) (Biomar LTD, Nersac, France). The daily ration was adjusted to water temperature in accordance with the manufacturer’s recommendations. Fish were unfed for 48 h prior to data collection. Dissolved oxygen, temperature, and pH were recorded twice daily using HACH HQ 40 multimeter. Temperature, pH value, and oxygen saturation is presented and ranged within the optimal level for brook trout (Figure 2).

### 2.2. Data Collection and Sample Analysis

On days 28, 56, 84, 112, and 140, 30 females and 30 males from each tank (*n* = 90 females and *n* = 90 males per group) were anaesthetized with clove oil (0.03 mL L^−1^) for body weight (BW) and total length (L_T_) measurement using Ohaus Scout SKX balance (0.1 g accuracy) (Ohaus corporation, Parsippany, NJ, USA) and ruler. On days 84, 112, and 140 30 males and 30 females (10 per tank) from each treatment plus control were killed with an overdose of anesthetic. Internal organs and gonads were removed and fixed in Bouin’s solution for later measurement using OHAUS Pioneer balance (0.01 g accuracy) and calculation of:

Hepatosomatic index (HSI) = 100 × liver weight/BW;

Gonadosomatic index (GSI) = 100 × gonad weight/BW;

Perivisceral fat index (PVSI) = 100 × fat weight/BW;

Condition factor (K) = (BW/L^3^) × 100;

Fillet yield (FY) = 100/BW × fillet weight;

Feed conversion ratio (FCR) = (TFS/WG), where TFS—total feed supplied (g), and WG—weight gain;

Specific growth rate (SGR) = ((lnBWf − lnBWi)/Nd) × 100, where lnBWf—natural logarithm of final body weight, lnBWi—natural logarithm of initial body weight, and Nd—number of feeding days.

Blood samples were collected from 30 males and 30 females of each group on days 84, 112, and 140. Plasma glucose was measured using kit (B3L8 × 7 G3-5375/R02, Abbott, IL, USA) according to the company’s protocol using Abbott Architect c8000 clinical chemistry analyzer (Abbott Laboratories, Abbott Park, IL, USA). Oestradiol and testosterone were analyzed by solid-phase, enzyme-labeled chemiluminescent competitive immunoassays using kit L2E2Z for oestradiol and kit L2M1Z for testosterone according to the company’s protocol using the Immulite 2000XPi immunoassay system (Siemens Healthcare GmbH, Erlangen, Germany). The volume of plasma samples was 2 µL for glucose, 25 µL for oestradiol, and 20 µL for testosterone. Analyses were carried out at Stafila laboratory, České Budějovice, Czech Republic. Male and female fish data were analyzed separately [11].

At the end of the trial, the fish were evaluated for the development of gonads and secretion of sperm and eggs by manual expression of gametes.

Survival rate was calculated using the following equation:

Survival (S) = 100 × Nf (Ni − Ns)^−1^,

where Ni and Nf: initial and final number of fish per tank, Ns: number of sampled fish per tank.

The costs for the production of 1 kg of fish (€ per kg fish) also were calculated:

PC = (DP + EC)/B,

where PC: production costs (€ per kg fish), DP: diet price (€/kg), EC: cost of energy (€), and B: increase in biomass over the period of the experiment (kg).

### 2.3. Statistical Analysis

Data were analyzed with Microsoft Excel 2010 (Microsoft, Inc., Redmond, Washington, DC, USA). Statistical analysis consisted of a one-way analysis of variance (ANOVA) followed by Tukey’s post-hoc test (Statistica 12.0; StatSoft, Inc., Tulsa, OK, USA). When the ANOVA assumptions were not satisfied, the differences between groups were tested using the nonparametric Kruskal–Wallis test. During statistical analyzing a Bonferroni correction was applied to adjust the *p*-values. The level of significance for all analyses was *p* < 0.05.

## 3. Results

**Figure 3:**
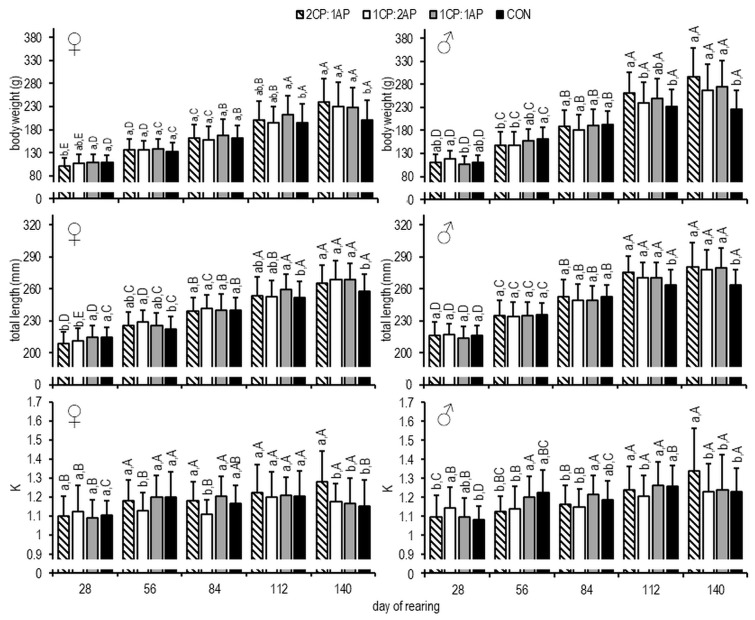
Brook trout body weight (BW, g), total length (L_T_, mm), and condition factor (K) after exposure to three non-circadian photoperiods (see Figure 1 for details). Data are mean (bars) ± SD—standard deviation (whiskers). Different small letters above bars indicate significant difference among treatments at a sampling date (*p* < 0.05) and different big letters indicate significant difference within the experiment group at a different sampling date (*p* < 0.05). 2CP:1AP—group exposed to 24 h of ambient photoperiod alternating with a 48 h of continuous light, 1CP:2AP—group exposed to 48 h of ambient photoperiod alternating with a 24 h of continuous light, 1CP:1AP—group exposed to 24 h of ambient photoperiod alternating with 24 h of continuous light, and CON—group exposed to natural ambient photoperiod as a control.

At the end of the growth experiment, the CON group showed significantly lower mean BW in both sexes females, 199.8 ± 12.2 g, and males, 226.6 ± 39.8 g, compared to the experimental groups (Figure 3).

The same was observed for L_T_, with the CON group having a lower mean length compared to all experimental groups (*p* < 0.05), regardless of sex (females, 258 ± 15 mmm and males, 264 ± 14 mm (Figure 3)).

**Figure 4:**
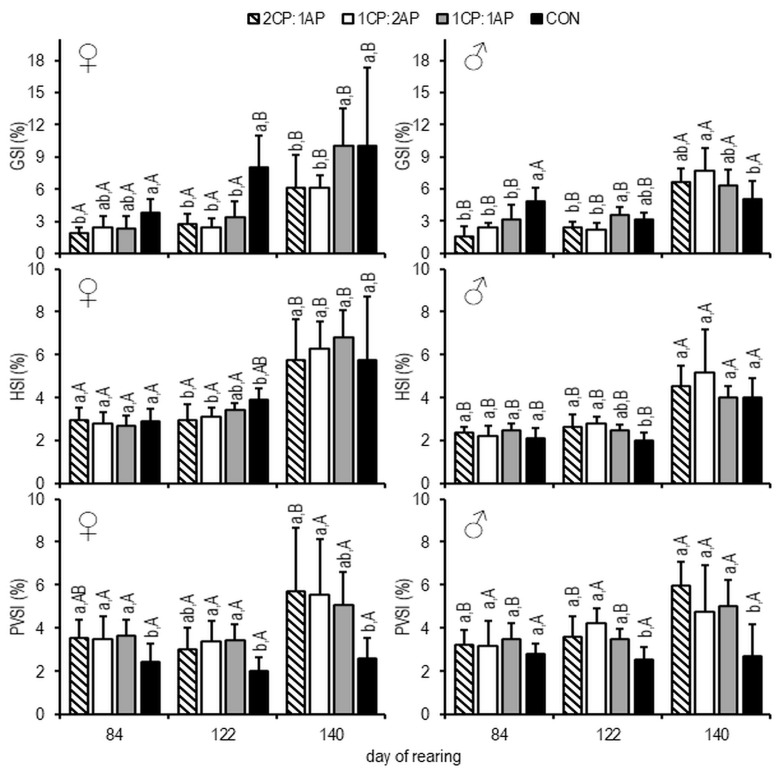
Hepatosomatic index (HSI, %), gonadosomatic index (GSI, %), and perivisceral fat index (PVSI, %) of brook trout reared under three non-circadian photoperiods (see Figure 1 for details). Data are mean (bars) ± SD—standard deviation (whiskers). Different small letters above bars indicate significant difference among treatments at a sample date (*p* < 0.05) and different big letters indicate significant difference within the experiment group at a different sampling date (*p* < 0.05). 2CP:1AP—group exposed to 24 h of ambient photoperiod alternating with a 48 h of continuous light, 1CP:2AP—group exposed to 48 h of ambient photoperiod alternating with a 24 h of continuous light, 1CP:1AP—group exposed to 24 h of ambient photoperiod alternating with 24 h of continuous light, CON—group exposed to natural ambient photoperiod as a control.

On day 84, the mean GSI of females was significantly higher in the CON group (3.75 ± 1.31) than in 2CP:1AP and, on day 112, compared to other experimental treatments (7.99 ± 3.02) (Figure 4). At the end of the experiment, females with lowest GSI were found in CP:1AP and 1CP:2AP groups (*p* < 0.05) (Figure 4).

The CON males showed significantly higher mean GSI compared experimental groups on day 84, and lower mean GSI than in the 1CP:2AP group on day 140 (*p* < 0.05) (Figure 4). The mean HSI of females was significantly higher in the CON group compared with 2CP:1AP and 1CP:2AP on day 112 (3.89 ± 0.53) (Figure 4). The opposite was observed in males, in which HSI of controls was lower than in the treatment groups on day 112 (*p* < 0.05) (Figure 4). Both sexes showed significantly lower PVSI in the CON group throughout the experiment (Figure 4).

**Figure 5:**
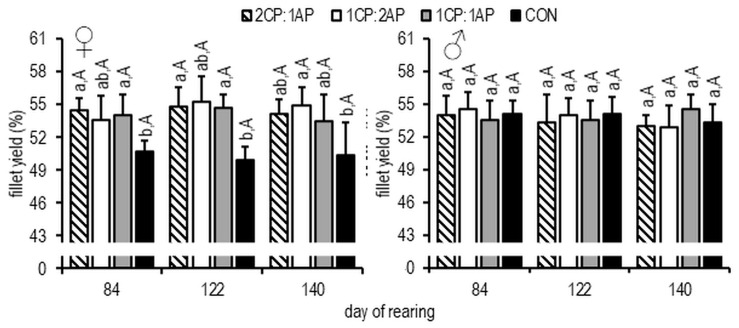
Fillet yield (%) of brook trout reared under three non-circadian photoperiods (see Figure 1 for details). Data are mean (bars) ± SD—standard deviation (whiskers). Different small letters indicate significant difference among treatments at a sample date (*p* < 0.05) and different big letters indicate significant difference within the experiment group at a different sampling date (*p* < 0.05). 2CP:1AP—group exposed to 24 h of ambient photoperiod alternating with a 48 h of continuous light, 1CP:2AP—group exposed to 48 h of ambient photoperiod alternating with a 24 h of continuous light, 1CP:1AP—group exposed to 24 h of ambient photoperiod alternating with 24 h of continuous light, CON—group exposed to natural ambient photoperiod as a control.

On day 84, the mean K (condition factor) of females was significantly lower in the 1CP:2AP group (1.11 ± 0.08), and, at the end of the study, the 2CP:1AP presented a higher K (1.28 ± 0.16) than in all other treatments and controls (*p* < 0.05) (Figure 3). The same results at the end of the growth experiment were found for the 2CP:1AP male group (Figure 2). At the end of the experimental trial, the fillet yield was significantly higher in the male 1CP:1AP group compared to all experimental groups (Figure 5). Additionally, FCR (feed conversion ratio) was analyzed during the experiment. There were no significant differences and, accordingly, FCR was: 2CP:1AP—1.25 ± 0.06, 1CP:2AP—1.27 ± 0.06, 1CP:1AP—1.23 ± 0.03, and CON—1.32 ± 0.02. In females, significantly reduced SGR (specific growth rate) (0.43 ± 0.09%/day^−1^) was found in the control group when compared to the 2CP:1AP group (0.62 ± 0.03%/day^−1^). Groups CP:2AP and 1CP:1AP showed similar values for SGR (0.55 ± 0.06%/day^−1^ and 0.53 ± 0.02%/day^−1^, respectively). Males SGR was also significantly reduced in control group (0.52 ± 0.05%/day^−1^) when compare to all other experimental groups. SGR reached 0.69 ± 0.08, 0.63 ± 0.04, and 0.67 ± 0.04%/day^−1^ in the 2CP:1AP, 1CP:2AP, and 1CP:1AP group, respectively.

**Figure 6:**
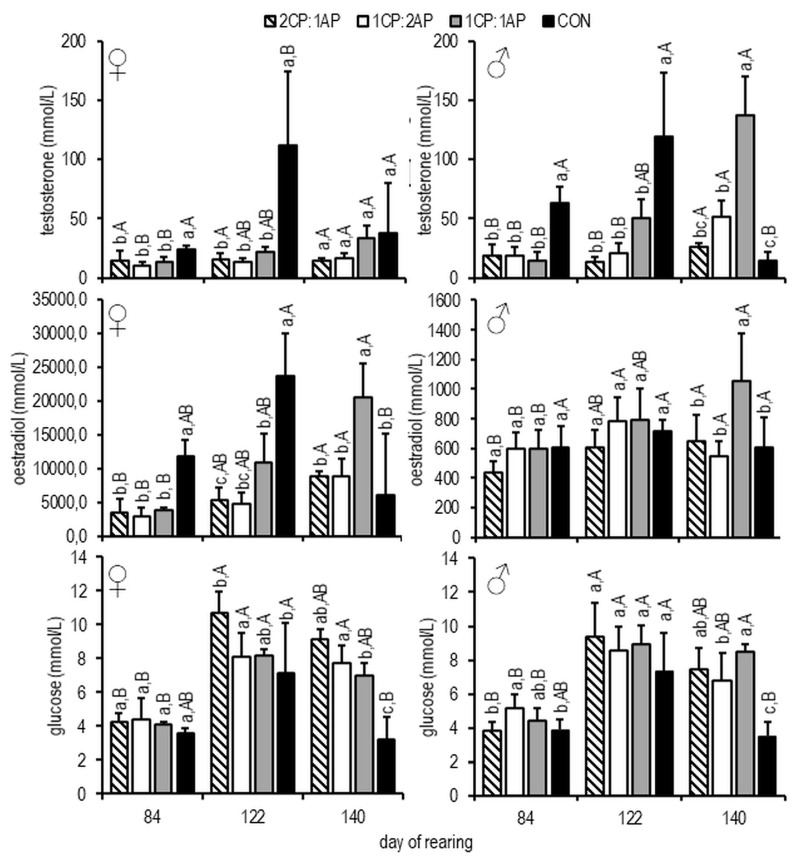
Glucose, testosterone, and oestradiol concentrations in the blood plasma of female and male brook trout reared under three non-circadian photoperiods (see Figure 1 for details). Data are mean (bars) ± SD—standard deviation (whiskers). Different letters indicate significant difference among treatments at a sample date (*p* < 0.05). 2CP:1AP—group exposed to 24 h of ambient photoperiod alternating with a 48 h of continuous light, 1CP:2AP—group exposed to 48 h of ambient photoperiod alternating with a 24 h of continuous light, 1CP:1AP—group exposed to 24 h of ambient photoperiod alternating with 24 h of continuous light, CON—group exposed to natural ambient photoperiod as a control.

On days 84 and 112, testosterone was higher in CON females and males than in the experimental groups (*p* < 0.05) (Figure 6). On day 140, the highest testosterone levels were observed in 1CP:1AP males, and highest oestradiol in 1CP:1AP females (Figure 6). A similar pattern was observed for oestradiol among females, where at the end of the study oestradiol was significantly higher in 1CP:1AP (Figure 6). Similarly, changes in glucose were observed regardless of sex. On day 112, glucose was higher in 1CP:2AP than in CON and 2CP:1AP in both females and males. At the end of the experiment CON showed the lowest levels of glucose among treatments (*p* < 0.05) (Figure 6).

**Figure 7:**
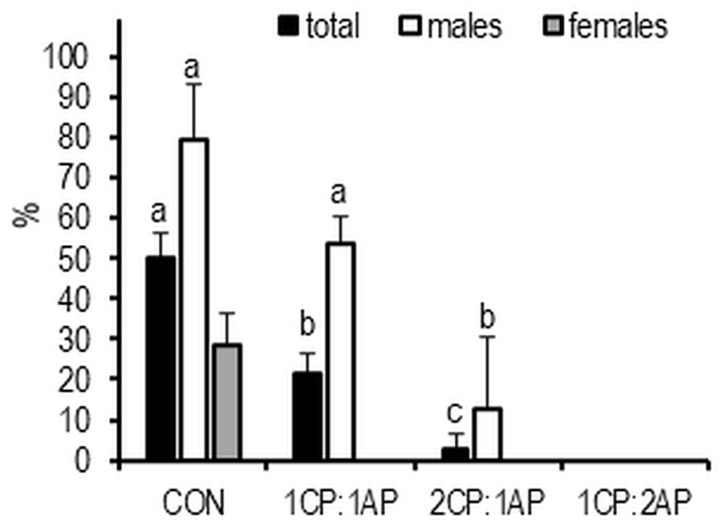
Spawning readiness of brook trout reared under three non-circadian photoperiods (see Figure 1 for details). Data are mean (bars) ± SD—standard deviation (whiskers). Different letters indicate significant difference among treatments at a sample date (*p* < 0.05). 2CP:1AP—group exposed to 24 h of ambient photoperiod alternating with a 48 h of continuous light, 1CP:2AP—group exposed to 48 h of ambient photoperiod alternating with a 24 h of continuous light, 1CP:1AP—group exposed to 24 h of ambient photoperiod alternating with 24 h of continuous light, CON—group exposed to natural ambient photoperiod as a control.

The highest proportion of mature fish, based on abdominal palpation, was 80% in CON males and 29% in CON females. A significantly lower rate of mature males was observed in 1CP:1AP and 2CP:1AP groups (54% and 13%, respectively). No fish from the 1CP:2AP group matured (Figure 7). At the end of the experimental trial, survival was calculated and no statistical differences were found. The lowest survival was observed in the control group—89.7 ± 1.7, and the highest in 1CP:2AP group—92.2 ± 1.0, but no significant differences were observed.

The cost of maintaining the prolonged photoperiods was €25 for 1CP:2AP, €37 for 1CP:1AP, and €49 for 2CP:1AP. Final calculations, including all expenses, revealed a cost kg^−1^ biomass of €7.7 ± 0.84 for CON group, €7.8 ± 0.43 for 1CP:1AP, €8.7 ± 0.35 for 2CP:1AP, and €7.4 ± 1.12 for 1CP:2AP.

## 4. Discussion and Conclusions

We found significant effects of a prolonged photoperiod on the growth rate and time of puberty with both male and female controls showing significantly higher levels of preparedness for spawning, suggesting use of the non-circadian photoperiod regime as an effective option in commercial fish culture.

The photoperiod is widely recognized as the major determining factor in sexual maturation of salmonids [28]. The seasonal timing of salmonid spawning can be manipulated with constant short or long photoperiods [29] and by condensing or extending the annual photoperiod cycle [18,30]. Moreover, factors such as temperature, photoperiod manipulation, and feeding strategy are able to delay sexual maturity to some degree [14]. However, there is limited information about the non-circadian photoperiod on development and growth.

Organisms reared under non-circadian cycles may suffer effects of disturbance to physiological processes [31]. Dalley [31] suggested that exposure to environmental cycles with non-circadian periods might be beneficial to prawns, as life-history processes can be accelerated via compression of the organism’s relative time-scale. Von Saint Paul and Aschoff [32] and Saunders [33] stated that non-circadian photoperiods exert a negative effect on growth and survival of some insects. It has been reported that rearing under non-circadian light regimes can cause increased mortality, reductions in growth, and developmental changes in the prawn *Palaemon elegans* [34]. Results of our study demonstrate a growth-promoting effect of non-circadian photoperiod regimes.

The chief role of sex steroids is the regulation of gametogenesis via the synthesis of vitellogenin and the proliferation of spermatogonia during spermatogenesis [35]. We observed significantly higher testosterone and oestradiol plasma levels in male and female CON, with a lower level of these sex steroids levels in experimental groups, indicating a delay of puberty. A sharp decrease in the level of testosterone and oestradiol was seen over the course of the experiment. Studies of salmonids report a reduction and return to baseline concentrations in oestradiol immediately before ovulation [36,37].

Our finding of significantly higher oestradiol in females than in males is consistent with previous studies [38]. Testosterone’s effect on gonadotropin production and its role as a precursor to estrogens explain elevated plasma testosterone in females [39]. Analyses of testosterone and its changes in males are in agreement with reported results for brook trout and other salmonids [3,6,35].

Our data of growth with respect to photoperiod manipulation of brook trout are in agreement with studies of sex steroid production in rainbow trout (*Oncorhynchus mykiss*) and Atlantic salmon [40,41]. Sex steroids are known as regulators of nutrient partitioning, especially in mammals. However, there are various responses in steroid among fish [42]. For example, in salmonids testosterone and estradiol-17β upregulate and downregulate [43]. Estradiol-17β regulates lipogenic genes in liver [44] and increases protein turnover [45]. Moreover, in salmonids muscle estradiol-17β and testosterone regulate sensitivity to hormone/insulin-like growth factor and other growth-related mechanisms [42]. Summarizing the known results, it can be assumed that in salmonids estrogenic compounds affect physiological mechanisms in many tissues, which causes the redistribution of nutrients from muscle building to supporting gonadal development [42]. The obtained results of this study showed that the growth rate of fish reared under artificially adjusted light regimes was higher than those reared under ambient light. The effects of prolonged light regimes on growth during the typical spawning period can be explained by suppression of maturation and allocation of energy to somatic growth as opposed to gonad development, which was confirmed by the observed among-group GSI differences during the spawning period, which were most pronounced in males. The male CON group showed increased GSI during the prespawning period, with a decrease after spawning. For a limited period, the GSI of the experimental groups began to increase. A similar trend was observed among females. This observation may indicate a process of delayed puberty in brook trout due to the influence of non-circadian photoperiod regimes. Rather similar results of the photoperiod influence and its reflection on GSI were published by Noori et al. [46], where they demonstrated that a long-day photoperiods 24L:0D and 18L:6D can affect the growth and delay the gonadal development of rainbow trout. In agreement with Randall et al. [47] and Taylor et al. [48], growth stimulation in the present study was not associated with the extended light period, but by an increase of daylight hours.

During the study, fish were monitored for spawning preparation. The results of spawning readiness obtained at the conclusion of the trial showed that a statistically higher percentage of fish from control groups were matured. It should be noted that a significantly larger number of matured individuals were males between control groups, and there were no matured females in experimental treatments. These results confirm the concept that males mature earlier than females [4]. It has been suggested that the initiation of sexual maturation is linked to a rate of accumulation of lipid stores and their absolute levels [49]. This may explain the late onset of puberty in females because the fecundity of females and offspring survival are enhanced by increases in energy reserves [50].

We conclude that fish maturation is delayed by a prolonged photoperiod under non-circadian regimens, as indicated by the data of GSI, sex steroid levels, and spawning readiness of fish during the natural spawning period. These results can be compared with those of previous studies revealing that several photoperiodic species undergo spontaneous gonad regression during rearing under stimulatory day lengths [51,52] and show changes in responsiveness to long daylight periods that do not reach the length of the experimental stimulating day length [53].

We successfully applied non-circadian photoperiods to improve the growth performance of brook trout, regardless of sex, by delaying sexual maturation. Lower production costs can be achieved by employing the technique for as short a period as necessary. Our period of 46 days of artificial lighting during 140 rearing days showed a positive effect.

## Data Availability

The data that support the findings of this study are available from the corresponding author upon reasonable request.
